# Increased time from physiological derangement to critical care admission associates with mortality

**DOI:** 10.1186/s13054-021-03650-1

**Published:** 2021-06-30

**Authors:** Stephen F. Whebell, Emma J. Prower, Joe Zhang, Megan Pontin, David Grant, Andrew T. Jones, Guy W. Glover

**Affiliations:** 1 Department of Critical Care, Guys and St Thomas NHS Foundation Trust, Westminster Bridge Road, London, SE1 7EH UK; 2 Department of Critical Care, Kings College Hospital, Denmark Hill, London, SE5 9RS UK; 3 Department of Quality and Assurance, Guy’s and St Thomas NHS Foundation Trust, Westminster Bridge Road, London, SE1 7EH UK; 4 Department of Clinical Informatics, Guys and St Thomas NHS Foundation Trust, Westminster Bridge Road, London, SE1 7EH UK

**Keywords:** Critical care, Clinical deterioration, Hospital rapid response team, Intensive care unit, Organisation and administration, Early warning scores

## Abstract

**Background:**

Rapid response systems aim to achieve a timely response to the deteriorating patient; however, the existing literature varies on whether timing of escalation directly affects patient outcomes. Prior studies have been limited to using ‘decision to admit’ to critical care, or arrival in the emergency department as ‘time zero’, rather than the onset of physiological deterioration. The aim of this study is to establish if duration of abnormal physiology prior to critical care admission [‘Score to Door’ (STD) time] impacts on patient outcomes.

**Methods:**

A retrospective cross-sectional analysis of data from pooled electronic medical records from a multi-site academic hospital was performed. All unplanned adult admissions to critical care from the ward with persistent physiological derangement [defined as sustained high National Early Warning Score (NEWS) > / = 7 that did not decrease below 5] were eligible for inclusion. The primary outcome was critical care mortality. Secondary outcomes were length of critical care admission and hospital mortality. The impact of STD time was adjusted for patient factors (demographics, sickness severity, frailty, and co-morbidity) and logistic factors (timing of high NEWS, and out of hours status) utilising logistic and linear regression models.

**Results:**

Six hundred and thirty-two patients were included over the 4-year study period, 16.3% died in critical care. STD time demonstrated a small but significant association with critical care mortality [adjusted odds ratio of 1.02 (95% CI 1.0–1.04, *p* = 0.01)]. It was also associated with hospital mortality (adjusted OR 1.02, 95% CI 1.0–1.04, *p* = 0.026), and critical care length of stay. Each hour from onset of physiological derangement increased critical care length of stay by 1.2%. STD time was influenced by the initial NEWS, but not by logistic factors such as out-of-hours status, or pre-existing patient factors such as co-morbidity or frailty.

**Conclusion:**

In a strictly defined population of high NEWS patients, the time from onset of sustained physiological derangement to critical care admission was associated with increased critical care and hospital mortality. If corroborated in further studies, this cohort definition could be utilised alongside the ‘Score to Door’ concept as a clinical indicator within rapid response systems.

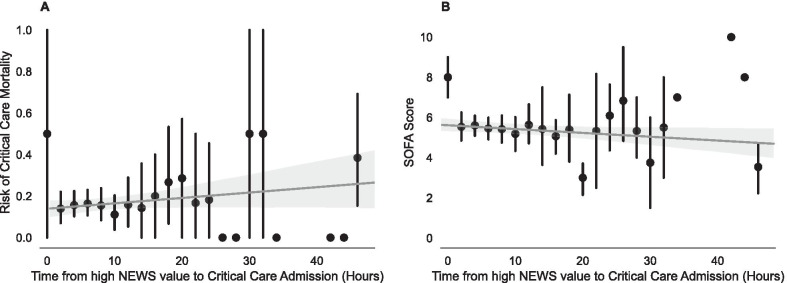

**Supplementary Information:**

The online version contains supplementary material available at 10.1186/s13054-021-03650-1.

## Introduction

The majority of unexpected hospital cardiac arrests, deaths, or unplanned critical care admissions are preceded by a trajectory of physiological deterioration [1–4], and it is suggested that these events can be anticipated, and potentially prevented. Whilst the development and utility of early warning scores have been criticised [5–7], rapid response systems have been associated with improvements in outcome [8, 9]. Specifically, the National Early Warning Score (NEWS) is an established system for detection of the deteriorating patient, (based upon deviation from normal physiology in 6 parameters; respiratory rate; SpO_2_; systolic blood pressure; heart rate; level of consciousness and temperature, with additional weighting for supplemental oxygen) in use across the United Kingdom (UK) National Health Service (NHS) since 2012. It was designed to standardise grading of acute illness severity using an ordinal scale from zero to 20 and has been shown to be predictive of adverse outcomes [10–12].

In 2011, the ‘score to door time’ [13] was proposed as a benchmarking tool to assess timeliness of the response to deterioration, and subsequent admission to critical care. The authors identified that organisational as well as clinical factors may influence the timeliness of admission; however, the relationship with clinical outcomes was not explored.

A number of studies have demonstrated that timely critical care outreach team (CCOT) response [14] and prompt admission to critical care [15–17] are associated with improvements in patient outcome whilst other studies have failed to show this relationship [18, 19]. There has been an imperfect definition of ‘time zero’, with studies commonly using the time of decision to admit (DTA) to critical care [15, 18, 20] rather than the onset of physiological deterioration and several studies have focused solely on the Emergency Department (ED) [15, 16, 21]. Furthermore, there are multiple confounders that may influence both time to admission and patient outcomes, including critical care capacity [17, 20], chronic health and frailty [22], surgical versus medical status [19], and acute sickness severity [13, 18] and these may account for the heterogeneity in the literature. Adjustment for differences in patient characteristics significantly alter the apparent relationship between timeliness and outcome [20]. To date, no studies have explored time from onset of physiological deterioration in ward patients to critical care admission, as a predictor of outcome.

Despite this, in 2019, the UK NHS utilised the Commissioning for Quality and Innovation (CQUIN) mechanism to focus a quality improvement effort on more timely response to deterioration on wards and admission to critical care, with the intended benefit of reducing critical care length of stay and hospital mortality [23]. For this metric, the start of the pathway was not clearly defined, which may lead to problematic heterogeneity.

## Objectives

We aim to describe a derived cohort of unplanned admissions to critical care with a high level of plausibility to benefit from prompt admission, and to test the association between duration of physiological derangement with subsequent patient outcomes. Additionally, we aim to explore factors associated with the Score to Door time.

## Methods

### Study design and setting

A retrospective study was conducted using unplanned admissions to critical care in two hospitals at a single academic medical centre [Guys’ and St Thomas’ Foundation Trust, (GSTT)] between 1/1/2017 and 1/03/2021.

The hospitals have a fully deployed NEWS protocol, utilising observations recorded in real time into an electronic health record. Observation frequency was protocolised in accordance with NEWS [10]. The system incorporates advisory notifications to encourage adherence to the escalation policy, and a population level report that allowed oversight of all high NEWS patients across the hospital, but without specific electronic alerts of high NEWS to caregivers [24].

In accordance with the NEWS protocol, patients with a NEWS of 5 or 6 should trigger an urgent assessment by a clinician with acute care skills, whilst a NEWS of greater than, or equal to 7 should trigger an emergency response by a team with critical care skills (the CCOT).

A 24/7 CCOT was present, staffed by advanced nurse practitioners with dedicated middle and senior grade critical care doctors. Escalation to the CCOT was required for any admission to critical care and admission decisions were made by the attending critical care consultant. The critical care service compromised a 72 bedded, mixed level 2/3 service. During the COVID-19 pandemic, this service underwent expansion up to 210 beds with additional surge staffing.

Non-invasive ventilation (NIV) was only provided in critical care areas, whilst high flow nasal cannula oxygen could be delivered in selected ward areas with the support of trained ward teams and/or the CCOT. Additional protocols for the urgent management of sepsis [25] and acute kidney injury [26] on the wards were present.

### Study population

We included adult patients with unplanned admissions to critical care from the ward, whom prior to admission had new and sustained physiological derangement. This was defined as the first occurrence of a NEWS greater than or equal to 7 that did not subsequently decrease below 5 in the interval prior to critical care admission. This definition was utilised as it aligns with existing NEWS trigger thresholds, whilst including a degree of tolerance in recognition that physiological parameters may naturally vary and/or there may be a partial response to treatment. Unplanned admission was defined as admission to a level 2 or 3 critical care unit from a level 1 ward, without an intervening operating theatre visit. A convenience sample was used from the onset of our study database to present. Patients were excluded if they were admitted to critical care directly from the ED or if time between initial high NEWS and critical care admission was > 7 days.

### Exposures and outcomes

The primary exposure was time from initial high NEWS of greater than or equal to seven, to critical care admission (‘Score to Door’ time). Time of critical care admission was defined as the time of the first recorded heart rate, to avoid errors associated with administrative processes. The primary outcome was mortality during the first critical care admission following initial fulfilment of study inclusion criteria. Secondary outcomes were length of index critical care admission and hospital mortality, which may have included death during subsequent critical care admissions.

### Data collection

Data were extracted from the GSTT Data Warehouse, which serves as an aggregate repository of data from multiple electronic sources, developed using the Health Catalyst® Data Operating System (DOS™) (Health Catalyst, Inc. Salt Lake City, Utah). Sickness severity was estimated using the sequential organ failure assessment (SOFA) [27] on day one of the critical care admission. Co-morbidity was assessed using the Charlson–Deyo co-morbidity index [28] and frailty was assessed using the Dr Foster Global Frailty Score [29]. Out-of-hours was defined as 00:00 Saturday to 00:00 Monday, and 20:00–07.59 on weekdays. Block descriptions for primary ICD-10 codes were used to categorise primary diagnoses. Cases with sepsis were identified by ICD-10 codes A40/A41 [30].

### Statistical analysis

Continuous variables are presented with median and interquartile range and categorical variables with count and percentage. Between-group comparisons are made using the Mann–Whitney U test for continuous and Fisher’s Exact test for binary categorical variables. Patients with missing data in any variables of interest were excluded, resulting in no missing data.

The association between potential predictor variables and both critical care and hospital mortality were assessed using logistic regression. Logistic models were constructed in a stepwise fashion. Significant variables were determined by univariable logistic regression at *p* < 0.2. Significant variables were then further assessed in a multivariable logistic regression model that included Score to Door time.

Multivariable linear regression was used to explore the relationship between potential predictor variables and Score to Door time, as well as critical care length of stay. Variables were chosen based on their plausible contribution to the target variable. Dependent variables underwent natural log transformation due to their skewed distribution.

A sensitivity analysis was performed by repeating the final multivariable models on a dataset with missing data imputed rather than excluded. Imputation was performed by replacing missing values with the median of the available data for each variable.

Confidence intervals for multivariable models were calculated using the bias-corrected accelerated bootstrap method. A threshold of *p* < 0.05 was used to determine statistical significance in final models and comparative tests. All data manipulation and analysis was  performed using Python 3.8 [31–34].

The study was registered locally and was conducted as a service evaluation as defined by the U.K. NHS Health Research Authority (http://www.hra.nhs.uk) using anonymous, routinely collected data and therefore did not require review by the Research Ethics Committee.

## Results

### Characteristics and outcomes

Between 1/1/17 and 1/3/21 there were 6549 unplanned admissions to critical care. 3271 were excluded due to direct admission from ED, 2563 were excluded as they did not meet criteria for sustained high NEWS and 83 were excluded due to missing data (summary of missing data presented in Additional file 1: Table 1). This resulted in 632 patients included in the final analysis (Flowchart in Additional file 1: Fig. 1). The characteristics and outcomes of analysed patients are presented in Table 1. Baseline demographics of survivors and decedents were similar, excepting a higher frailty score in patients who survived their index critical care admission.

**Table 1:** Characteristics and outcomes of the study population

	All (*n* = 632)	Survived critical care (*n* = 529)	Died critical care (*n* = 103)	*p*
*Demographics and co-morbidity scores*				
Age*	66.0 [54.0–76.0]	65.0 [53.0–75.0]	67.0 [56.0–78.0]	0.107
Male gender*	384 (60.8%)	322 (60.9%)	62 (60.2%)	0.912
Charlson–Deyo co-morbidity index*	10.0 [6.0–17.0]	11.0 [6.0–18.0]	10.0 [7.0–14.0]	0.453
Frailty score*	0.0 [0.0–2.8]	1.0 [0.0–2.8]	0.0 [0.0–2.8]	0.04
*Primary diagnosis group*^#^				
Respiratory	160 (25.3%)	138 (26.1%)	22 (21.4%)	0.386
Neoplasms	95 (15.0%)	72 (13.6%)	23 (22.3%)	0.034
Circulatory^$^	81 (12.8%)	72 (13.6%)	9 (8.7%)	0.2
Infectious diseases^^^	80 (12.7%)	64 (12.1%)	16 (15.5%)	0.333
Gastrointestinal	67 (10.6%)	60 (11.3%)	7 (6.8%)	0.22
Injury or poisoning	32 (5.1%)	24 (4.5%)	8 (7.8%)	0.215
Genitourinary^&^	19 (3.0%)	18 (3.4%)	1 (1.0%)	0.339
Musculoskeletal	14 (2.2%)	12 (2.3%)	2 (1.9%)	1.0
Endocrine and metabolic	12 (1.9%)	10 (1.9%)	2 (1.9%)	1.0
Other^~^	31 (4.9%)	31 (5.9%)	0 (0.0%)	0.005
*Hospital admission characteristics*				
Elective admission*	145 (22.9%)	124 (23.4%)	21 (20.4%)	0.608
Emergency admission*	450 (71.2%)	373 (70.5%)	77 (74.8%)	0.408
Other admission*	37 (5.9%)	32 (6.0%)	5 (4.9%)	0.819
Sepsis during admission*	225 (35.6%)	191 (36.1%)	34 (33.0%)	0.576
Hospital length of stay (days)	22.9 [12.4–41.3]	25.1 [13.9–44.9]	12.7 [6.7—25.9]	< 0.001
Hospital mortality	173 (27.4%)	70 (13.2%)	103 (100.0%)	–
*Score to door characteristics*				
Time from hospital admission to initial high NEWS (days)*	3.3 [1.1–10.3]	3.0 [1.0–8.8]	5.4 [1.9–15.3]	0.002
Initial high NEWS value*	8.0 [7.0–9.0]	8.0 [7.0–9.0]	8.0 [7.0–9.5]	0.438
Initial high NEWS occurred out of hours*	342 (54.1%)	285 (53.9%)	57 (55.3%)	0.829
Peri-arrest call prior to critical care admission*	69 (10.9%)	55 (10.4%)	14 (13.6%)	0.387
Initial high NEWS to critical care admission (hours)	6.3 [3.8–10.2]	6.2 [3.8–10.0]	6.7 [3.9–12.2]	0.297
*Critical care characteristics*				
Day 1 SOFA score*	5.0 [3.0–8.0]	5.0 [3.0–7.0]	7.0 [4.0–10.0]	< 0.001
Invasive mechanical ventilation	163 (25.8%)	124 (23.4%)	39 (37.9%)	0.003
Non-invasive ventilation	136 (21.5%)	106 (20.0%)	30 (29.1%)	0.049
Invasive or non-invasive ventilation*	270 (42.7%)	212 (40.1%)	58 (56.3%)	0.003
Critical care length of stay (days)	3.2 [1.7–6.6]	3.3 [1.7–6.2]	3.0 [1.4–8.9]	0.936
Critical care mortality	103 (16.3%)	–	–	–

*NEWS* national early warning score, *SOFA* sequential organ failure assessment

*Variable utilised in stepwise logistic regression construction for both critical care and hospital mortality

^#^Derived from ICD-10 block descriptions

^$^Including cardiac surgery

^^^Including sepsis

^&^Including renal failure

^~^Including neurological and non-neoplastic haematological disorders

### Score to Door time and critical care mortality

**Fig. 1:**
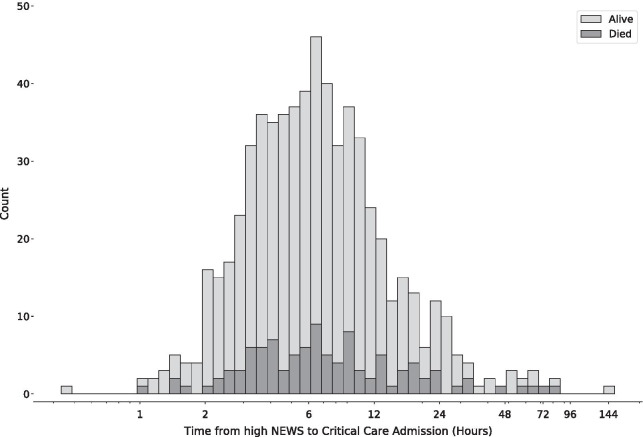
Distribution of time to critical care admission from initial high NEWS score (Score to Door time) (x-axis logarithmically scaled). *NEWS, national early warning score*

The median Score to Door time was 6.3 [3.8–10.2] hours with most patients admitted to critical care within 24 h of the onset of physiological derangement (Fig. 1). Variables utilised for stepwise model construction are indicated in Table 1, with the univariable results reported in Additional file 1: Table 2 and final multivariable models reported in Table 2.

**Table 2:** Multivariable logistic and log-linear regression models. Odds ratios presented for logistic models and coefficients presented for log-linear models

Variable	OR	Lower CI (0.025)	Upper CI (0.975)	*p*
*Multivariable logistic model for critical care mortality*				
Score to door time	1.02	1.0	1.04	0.01
Age	1.02	1.0	1.04	0.011
Dr Foster global frailty score	0.9	0.79	1.02	0.082
Charlson Deyo score	0.98	0.96	1.01	0.149
SOFA score	1.2	1.11	1.3	< 0.001
NIV or IMV	1.73	1.05	2.73	0.021
Time to triggering high NEWS	1.02	1.01	1.03	0.004
Constant	0.01	0.0	0.04	< 0.001
*Multivariable logistic model for hospital mortality*				
Score to door time	1.02	1.0	1.04	0.026
Age	1.02	1.01	1.03	0.001
Triggering high NEWS value	1.11	0.98	1.24	0.061
Time to triggering high NEWS	1.02	1.01	1.03	0.003
SOFA score	1.17	1.09	1.24	< 0.001
NIV or IMV	1.69	1.13	2.46	0.007
Elective hospital admission	0.64	0.4	1.04	0.056
Constant	0.01	0.0	0.04	< 0.001

Variable	*β*	Lower CI (0.025)	Upper CI (0.975)	*p*
*Multivariable log-linear model for critical care length of stay (survivors)*				
Score to door time	0.01	0.01	0.02	0.024
Age	0.0	− 0.01	0.01	0.467
Dr Foster global frailty score	0.01	− 0.03	0.06	0.761
Charlson–Deyo score	0.98	− 0.01	0.01	0.149
SOFA score	0.02	− 0.05	0.03	0.298
Sepsis status during admission	0.32	0.13	0.57	0.007
NIV or IMV	0.67	0.44	0.88	< 0.001
Constant	0.36	− 0.14	0.85	0.158
*Multivariable log-linear model for score to door time*				
Age	0.00	− 0.002	0.006	0.342
Dr Foster global frailty score	0.00	− 0.03	0.03	0.902
Charlson–Deyo score	0.00	− 0.005	0.006	0.853
Triggering high NEWS value	− 0.08	− 0.11	− 0.04	< 0.001
Time to triggering high NEWS	0.00	− 0.002	0.006	0.250
Triggering high NEWS out of hours	0.02	− 0.09	0.14	0.698
Peri-arrest call prior to critical care admission	− 0.07	− 0.25	0.14	0.479
Constant	2.39	1.99	2.79	< 0.001

OR, odds ratio; β, coefficient of variable in linear regression; CI, confidence interval; NIV, non-invasive ventilation; IMV, invasive mechanical ventilation; NEWS, national early warning score; SOFA, sequential organ failure assessment

Score to Door time demonstrated a small but significant association with critical care mortality with an unadjusted odds ratio of 1.02 (95% CI 1.0–1.03, *p* = 0.063) in a univariable model and an adjusted odds ratio of 1.02 (95% CI 1.0–1.04, *p* = 0.01) in the multivariable model. Age, SOFA score, need for NIV or invasive mechanical ventilation (IMV), and duration of hospital admission prior to triggering high NEWS also had a significant association with critical care mortality. Neither co-morbidity nor frailty had a statistically significant relationship.

**Fig. 2:**
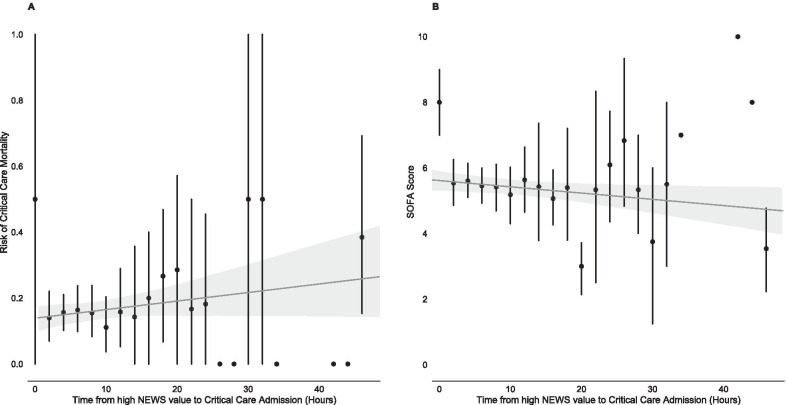
**A** Critical care mortality plotted against time from initial high NEWS score to critical care admission. **B** Admission SOFA score plotted against time from initial high NEWS score to critical care admission. Both plots divided into two hourly blocks with mean and 95% confidence intervals plotted. Single points represent blocks with only a single patient. Unadjusted linear regression ‘line of best fit’ with 95% confidence interval plotted in both **A** and **B**. Time from high NEWS score to critical care admission clipped at 48 h to more clearly demonstrate the majority of data. *NEWS, national early warning score; SOFA, sequential organ failure assessment*

In Fig. 2, Score to Door time is considered in 2 hourly blocks with mean critical care mortality and admission SOFA score plotted for each block. There is a trend to increasing critical care mortality with delay to admission despite a downward trend in day one SOFA score.

### Score to Door time and hospital mortality

The association with hospital mortality was also examined. Score to Door time had a significant association with hospital mortality (adjusted OR 1.02, 95% CI 1.0–1.04, *p* = 0.026). Age, SOFA score, need for NIV or IMV, and time to triggering high NEWS were also significantly associated with hospital mortality. Triggering high NEWS value and elective admission type did not reach statistical significance.

### Score to Door time and critical care length of stay

In patients who survived to discharge from critical care, a multivariable log-linear regression was constructed with critical care length of stay as the dependent variable (Table 2). Score to Door time was significantly associated with critical care length of stay, with each hour increasing length of stay by 1.2% (β = 0.012, 95% CI 0.01–0.02, *p* = 0.024). Use of NIV or IMV and sepsis status were also significantly associated. Age, frailty, co-morbidity and SOFA score were not significantly associated with critical care length of stay.

### Determinants of Score to Door time

**Fig. 3:**
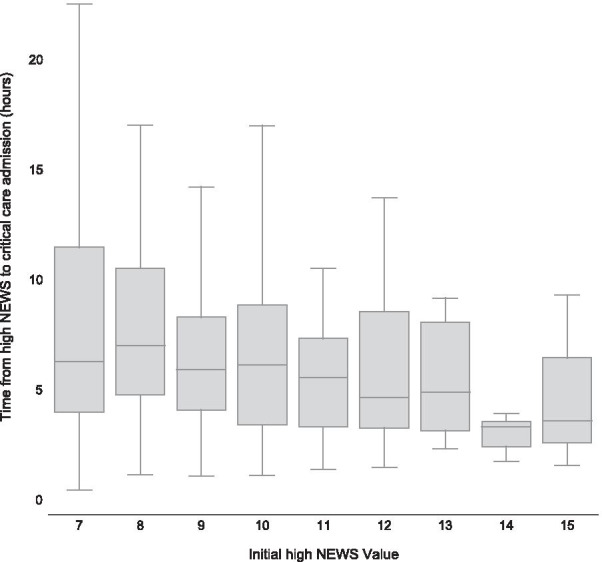
Boxplot of Score to Door time for initial high NEWS value. Whiskers 1.5 × IQR. *NEWS; national early warning score*; *IQR; inter-quartile range*

A multivariable log-linear regression was constructed with Score to Door time as the dependent variable (Table 2). The value of the triggering high NEWS was the only variable significantly associated with time to critical care admission with each point increment reducing the  time by 7.9% (β =  − 0.079, 95% CI − 0.11 to − 0.04, *p* = 0.007). This relationship is visualised in Fig. 3. Age, frailty, co-morbidity, time to high NEWS, out of hours triggering NEWS and peri-/cardiac arrest call prior to critical care did not demonstrate a significant relationship.

### Sensitivity analysis

Imputation of missing values did not change the level of significance of any of the covariates analysed in the multivariable logistic or linear models, with the detailed results presented in Additional file 1: Table 3.

## Discussion

In this study of 632 unplanned critical care admissions, and following adjustment for patient demographics, sickness severity, co-morbidity and frailty, the time from onset of sustained physiological deterioration to critical care admission was associated with a small but important increase in both critical care and hospital mortality, as well as a longer critical care length of stay. The Score to Door time was influenced by the triggering NEWS value, but not by logistic factors such as out-of-hours status, nor by patient factors such as co-morbidity or frailty.

### Comparison with the literature

Whilst it is intuitive that more rapid admission from wards to critical care following a high NEWS trigger will be beneficial, this is the first study to our knowledge to demonstrate this. The previous literature has either focused on patients in the ED [15, 16, 21], or has used the time of decision to admit [15, 18, 20]. Focussing solely on the efferent pathway ignores an important process of detection, escalation, response, and decision. Barriers to prompt recognition and escalation include a lack of timely or complete observations, poor communication, delayed response by CCOTs, or lack of senior decision maker [35], during which time the patient may suffer further deterioration [20]. We advocate for a whole-systems approach to improving both the afferent and efferent response to deterioration [36], and merely increasing critical care bed capacity will not necessarily address the challenges. Indeed, the benefit may be mediated by the prompt recognition, escalation and CCOT response, rather than critical care admission per se [14].

Whilst it is recommended to admit within 4 hours [21, 23], we found no particular inflection and it is likely that the increased risk is continuous if not linear.

### Strengths and weaknesses

Our study captures the entire period from the onset of deterioration to admission for definitive care. We utilised a strict definition of the cohort to include only patients who had sustained physiological abnormalities and with plausible potential to benefit from prompt admission. By doing so, we reduced heterogeneity before then adjusting for factors that may influence the timeliness of critical care admission, most notably sickness severity. This is important because it ought to be expected that sicker patients are admitted more quickly and this may confound the relationship with outcome [18, 20]. This study goes a step further than previous publications by proposing a clear definition of an at-risk cohort based on established NEWS thresholds that can be replicated in other studies and could be used for benchmarking. By examining the effect of logistic as well as clinical factors, our data provides some insights into the question of differential standards in out-of-hours care—an area of significant contention in healthcare [37].

However, we recognise certain limitations. This relatively small, single centre and retrospective study occurred in an academic institution with a relatively high ratio of critical care to ward level beds and as such, these findings cannot necessarily be generalised to other settings. We speculate that the magnitude of the effect would be greater in hospitals that have more limited access to critical care and where delays may be greater. Whilst the strictly defined cohort limits heterogeneity, it may also reduce generalisability and the findings cannot be extrapolated to all patients with elevated NEWS. It is noted that despite the selected cohort, the effect size is small.

We utilised critical care mortality as a primary outcome due to its plausible relationship to Score to Door time but we were unable to examine patient centred outcomes following discharge from hospital.

Inherent to the retrospective design, this study does not prove causality and there may be other factors that influence clinician or system behaviour with respect to escalation and response that are themselves associated with outcome and that are not captured by our co-variates. For example, some patients may have had baseline abnormal physiology (equating to ‘false positive’ NEWS triggers) or limitation of treatment orders that affected the Score to Door time and also outcome; furthermore we were unable to control for critical care capacity and organisational stress—delays occur when the system is under increased pressure and it may be this more general phenomenon that affects care and outcome [38]. Finally, we have only used surrogates of the start and end of the pathway and there may be inaccuracies in determining the true time of deterioration or critical care admission.

### Future directions

It is unknown if the relationship demonstrated exists across all strata of sickness severity or if it applies to alternative definitions of the target population.. This deserves further investigation, as patients with lower NEWS triggers are likely to be more numerous and experience longer waits to definitive treatment. The previous literature [20] has suggested that prompt admission may be less impactful for lower acuity critical care admissions and the association between timeliness of critical care admission and outcomes in a broader population of deteriorating patients should be validated before wholesale policy directives to improve care. Novel interpretations of NEWS over time, such as ‘area under the curve’ measurements, may aid in expanding the definition used in this paper to incorporate patients with lower absolute NEWS values. Finally, the impact of prolonged physiological derangement in patients ultimately not admitted to critical care also requires investigation, as this will aide in the development of future practice guidelines for managing acute deterioration.

## Conclusions

In a strictly defined population of high NEWS patients, the time from onset of sustained physiological derangement to critical care admission is associated with increased critical care and hospital mortality. If corroborated in larger studies, this would support the use of this population definition and the ‘Score to Door’ concept to standardise reporting and drive quality improvement initiatives in rapid response systems.

## Supplementary Information

**Additional file 1.** Additional figures and tables, referenced explicitly in text. Including flowchart of inclusion/exclusion and tabulated results from univariable regression models.

## Data Availability

All data generated or analysed during this study are included in this published article and its supplementary information files. The corresponding author may provide specified analyses or fully de-identified parts of the dataset upon reasonable request.

## Abbreviations

STD: Score to door time (hours from onset of physiological derangement to critical care admission)
NEWS: National early warning score
CCOT: Critical care outreach team
DTA: Decision to admit (to critical care)
CQUIN: Commissioning for quality and innovation
GSTT: Guys’ and St Thomas’ Foundation Trust
NIV: Non-invasive ventilation
HFNC: High flow nasal cannula
IMV: Invasive mechanical ventilation
SOFA: Sequential organ failure assessment
ED: Emergency department
