# Supplementary material for: Increased time from physiological derangement to critical care admission associates with mortality
Source: Crit Care. 2021 Jun 30;25:226. doi: 10.1186/s13054-021-03650-1 (PMC8243047; doi:10.1186/s13054-021-03650-1)
Supplement: Supplementary file 1 — Additional file 1. Additional figures and tables, referenced explicitly in text. Including flowchart of inclusion/exclusion and tabulated results from univariable regression models. [file 13054_2021_3650_MOESM1_ESM.docx]

**The time from physiological derangement to critical care admission associates with mortality**

*Supplementary Material*

Stephen F Whebell, Emma J Prower, Joe Zhang, Megan Pontin, David Grant, Andrew T Jones, Guy W Glover


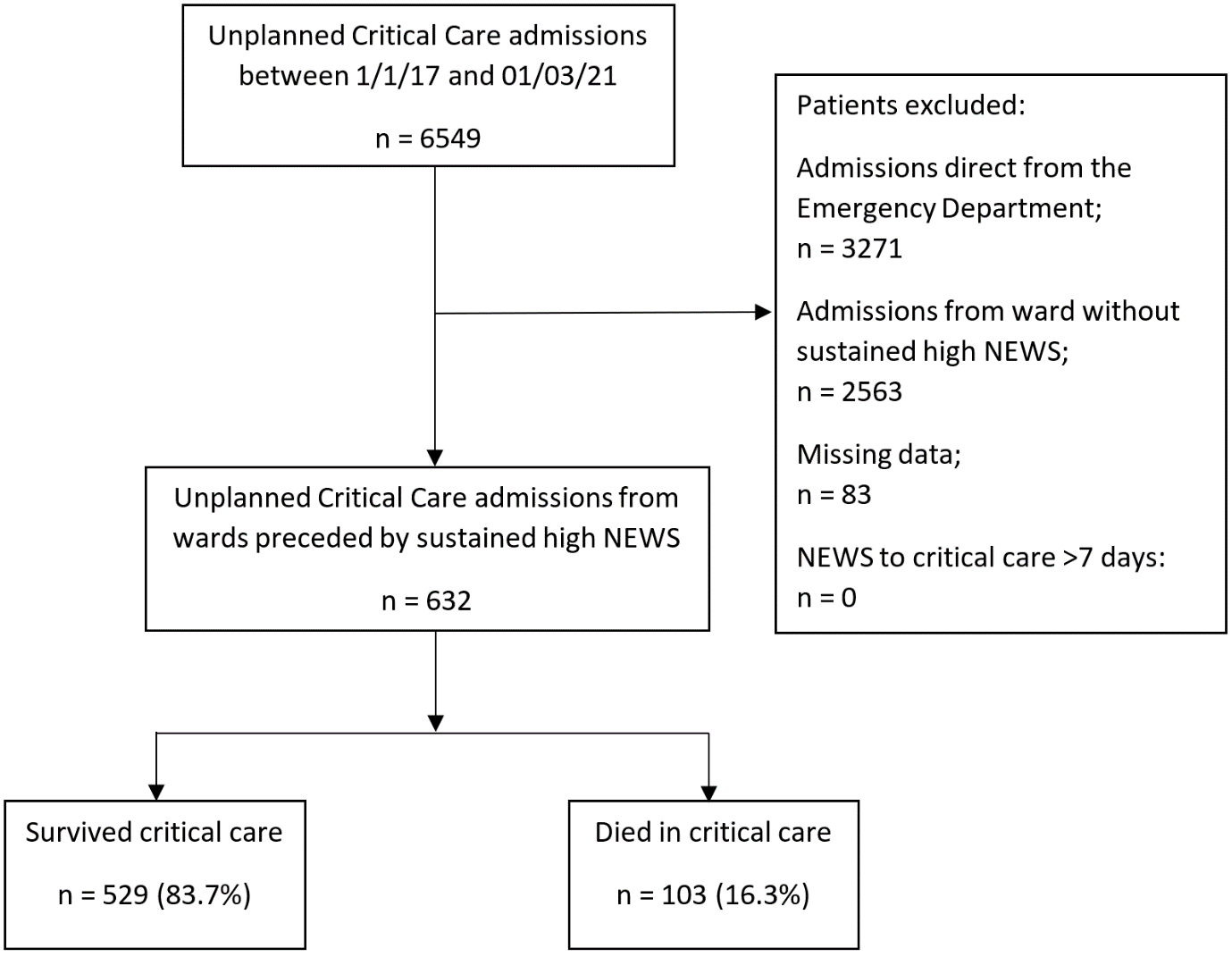


***Supplementary Figure 1:*** Flow chart of patient inclusion and exclusion

|  | **Full dataset**  **(n=715)** | | | **Dataset analysed**  **(n=632)** | |
| --- | --- | --- | --- | --- | --- |
|  | Number of observations | Percent of total observations | | Number of observations | Percent of total observations |
| Age | 661 | | 92.44% | 632 | 100.0% |
| Male gender | 715 | | 100.0% | 632 | 100.0% |
| Charlson-Deyo Comorbidity Index | 715 | | 100.0% | 632 | 100.0% |
| Frailty Score | 715 | | 100.0% | 632 | 100.0% |
| Primary diagnosis code | 715 | | 100.0% | 632 | 100.0% |
| Admission type | 661 | | 92.44% | 632 | 100.0% |
| Sepsis during admission | 715 | | 100.0% | 632 | 100.0% |
| Hospital length of stay | 690 | | 96.50% | 632 | 100.0% |
| Hospital mortality | 715 | | 100.0% | 632 | 100.0% |
| Time from hospital admission to initial high NEWS | 715 | | 100.0% | 632 | 100.0% |
| Initial high NEWS value | 715 | | 100.0% | 632 | 100.0% |
| Time of initial high NEWS | 715 | | 100.00% | 632 | 100.0% |
| Peri-arrest call prior to critical care admission | 715 | | 100.0% | 632 | 100.0% |
| Initial high NEWS to critical care admission | 715 | | 100.0% | 632 | 100.0% |
| Day 1 SOFA score | 632 | | 88.39% | 632 | 100.0% |
| Invasive Mechanical Ventilation | 715 | | 100.0% | 632 | 100.0% |
| Non-invasive Ventilation | 715 | | 100.0% | 632 | 100.0% |
| Critical Care length of stay | 707 | | 98.88% | 632 | 100.0% |
| Critical care mortality | 715 | | 100.0% | 632 | 100.0% |

**Supplementary Table 1:** Missing data in the full dataset and the dataset used for analysis.

NEWS, national early warning score; SOFA, sequential organ failure assessment; NIV, non-invasive ventilation; IMV, invasive mechanical ventilation.

| **Univariable logistic models for critical care mortality (Supplementary)** | | | | |
| --- | --- | --- | --- | --- |
| Variable | OR | Lower CI (0.025) | Upper CI (0.975) | p |
| Age* | 1.02 | 1.0 | 1.03 | 0.063 |
| Male gender | 0.92 | 0.63 | 1.5 | 0.9 |
| Frailty Score* | 0.89 | 0.8 | 0.99 | 0.033 |
| Charlson-Deyo Score* | 0.98 | 0.96 | 1.0 | 0.105 |
| Triggering high NEWS2 score | 1.06 | 0.94 | 1.2 | 0.322 |
| Time to triggering high NEWS2 score* | 1.01 | 1.0 | 1.02 | 0.06 |
| Triggering high NEWS2 score out of hours | 1.06 | 0.69 | 1.62 | 0.785 |
| Peri-arrest call prior to critical care admission | 1.36 | 0.72 | 2.54 | 0.343 |
| Score To Door time* | 1.02 | 1.0 | 1.03 | 0.06 |
| SOFA Score* | 1.2 | 1.13 | 1.29 | <0.001 |
| Sepsis flagged at critical care admission | 0.87 | 0.56 | 1.37 | 0.548 |
| NIV or IMV* | 1.93 | 1.26 | 2.95 | 0.002 |
| Elective admission | 0.84 | 0.5 | 1.4 | 0.5 |
| Emergency admission | 1.24 | 0.76 | 2.0 | 0.384 |
| Other admission | 0.79 | 0.3 | 2.1 | 0.637 |
| **Univariable logistic models for hospital mortality (Supplementary)** | | | | |
| Variable | OR | Lower CI (0.025) | Upper CI (0.975) | p |
| Age* | 1.02 | 1.01 | 1.03 | 0.002 |
| Male gender | 1.03 | 0.72 | 1.47 | 0.871 |
| Frailty Score | 1.01 | 0.93 | 1.09 | 0.811 |
| Charlson-Deyo Score | 0.99 | 0.98 | 1.01 | 0.397 |
| Triggering high NEWS2 score* | 1.11 | 1.00 | 1.23 | 0.045 |
| Time to triggering high NEWS2 score* | 1.01 | 1.00 | 1.02 | 0.027 |
| Triggering high NEWS2 score out of hours | 1.01 | 0.71 | 1.44 | 0.945 |
| Peri-arrest call prior to critical care admission | 1.27 | 0.74 | 2.19 | 0.374 |
| Score To Door time* | 1.01 | 0.99 | 1.03 | 0.154 |
| SOFA Score* | 1.17 | 1.11 | 1.24 | <0.001 |
| Sepsis flagged at critical care admission | 1.16 | 0.81 | 1.67 | 0.411 |
| NIV or IMV* | 1.85 | 1.30 | 2.64 | <0.001 |
| Elective admission* | 0.73 | 0.47 | 1.13 | 0.15 |
| Emergency admission | 1.26 | 0.85 | 1.87 | 0.252 |
| Other admission | 1.13 | 0.55 | 2.34 | 0.741 |

**Supplementary Table 2:** Odds ratios, confidence intervals and p-values for each variable analysed in a univariate logistic regression. Confidence intervals presented are raw and not bootstrapped. Univariable model constants not reported.

OR, odds ratio; CI, confidence interval, NEWS, national early warning score; SOFA, sequential organ failure assessment; NIV, non-invasive ventilation; IMV, invasive mechanical ventilation. *used in final multivariate model.

**Sensitivity analysis**

Missing datapoints were imputed with the median of available data for each variable analysed. Three patients were subsequently excluded for having a time from initial high NEWS to critical care admission of >7 days. This resulted in 713 patients available for analysis.

| **Multivariable logistic model for critical care mortality** | | | | |
| --- | --- | --- | --- | --- |
| Variable | OR | Lower CI (0.025) | Upper CI (0.975) | p |
| Score To Door time | 1.02 | 1.0 | 1.03 | 0.01 |
| Age | 1.02 | 1.0 | 1.03 | 0.022 |
| Dr Foster Global Frailty Score | 0.86 | 0.7 | 1.05 | 0.134 |
| Charlson Deyo Score | 0.99 | 0.97 | 1.01 | 0.264 |
| SOFA Score | 1.21 | 1.12 | 1.31 | <0.001 |
| NIV or IMV | 1.71 | 1.09 | 2.67 | 0.016 |
| Time to triggering high NEWS | 1.02 | 1.0 | 1.03 | 0.011 |
| Constant | 0.02 | 0.01 | 0.07 | <0.001 |
| **Multivariable logistic model for hospital mortality** | | | | |
| Variable | OR | Lower CI (0.025) | Upper CI (0.975) | p |
| Score To Door time | 1.01 | 1.0 | 1.03 | 0.048 |
| Age | 1.02 | 1.01 | 1.03 | 0.001 |
| Triggering high NEWS value | 1.11 | 0.99 | 1.23 | 0.047 |
| Time to triggering high NEWS | 1.01 | 1.0 | 1.02 | 0.023 |
| SOFA score | 1.17 | 1.1 | 1.25 | <0.001 |
| NIV or IMV | 1.62 | 1.11 | 2.3 | 0.009 |
| Elective hospital admission | 0.64 | 0.4 | 1.03 | 0.056 |
| Constant | 0.01 | 0.0 | 0.04 | <0.001 |
| **Multivariable log-linear model for critical care length of stay (survivors)** | | | | |
| Variable | β | Lower CI (0.025) | Upper CI (0.975) | p |
| Score To Door time | 0.01 | 0.0 | 0.02 | 0.008 |
| Age | 0.0 | -0.01 | 0.01 | 0.482 |
| Dr Foster Global Frailty Score | 0.02 | -0.03 | 0.06 | 0.578 |
| Charlson-Deyo Score | 0.0 | -0.01 | 0.01 | 0.506 |
| SOFA Score | 0.02 | -0.05 | 0.03 | 0.316 |
| Sepsis status during admission | 0.31 | 0.14 | 0.57 | 0.008 |
| NIV or IMV | 0.7 | 0.44 | 0.88 | <0.001 |
| Constant | 0.33 | -0.2 | 0.86 | 0.216 |
| **Multivariable log-linear model for Score to Door time** | | | | |
| Variable | β | Lower CI (0.025) | Upper CI (0.975) | p |
| Age | 0.0 | -0.002 | 0.006 | 0.335 |
| Dr Foster Global Frailty Score | 0.03 | -0.03 | 0.03 | 0.285 |
| Charlson-Deyo Score | 0.0 | -0.005 | 0.007 | 0.559 |
| Triggering high NEWS value | -0.08 | -0.11 | -0.04 | <0.001 |
| Time to triggering high NEWS | 0.00 | -0.002 | 0.006 | 0.230 |
| Triggering high NEWS out of hours | 0.01 | -0.09 | 0.14 | 0.929 |
| Peri-/arrest call prior to critical care admission | -0.07 | -0.25 | 0.14 | 0.489 |
| Constant | 2.41 | 2.0 | 2.83 | <0.001 |

**Supplementary Table 3:** Multivariable logistic and log-linear regression model results of imputed dataset (sensitivity analysis). Odds ratios presented for logistic models and coefficients presented for log-linear models.

OR, odds ratio; β, coefficient of variable in linear regression; CI, confidence interval; NIV, non-invasive ventilation; IMV, invasive mechanical ventilation; NEWS, national early warning score; SOFA, sequential organ failure assessment
